# Association Between Vitamin D Deficiency and the Incidence of Atrial Fibrillation: A Systematic Review and Meta-Analysis

**DOI:** 10.3390/biomedicines14071580

**Published:** 2026-07-15

**Authors:** Angelica Cersosimo, Lucio Teresi, Marco Valerio Mariani, Riccardo Rovelli, Nicola Pierucci, Vincenzo Mirco La Fazia, Chiara Camastra, Giovanni Fazio, Enrico Vizzardi

**Affiliations:** 1 Institute of Cardiology, Department of Medical and Surgical Specialities, Radiological Sciences, and Public Health, University of Brescia, Spedali Civili di Brescia, 25123 Brescia, Italy; riccardo.rovelli97@gmail.com (R.R.); vizzardi72@gmail.com (E.V.); 2 Department of Experimental and Clinical Medicine, Magna Graecia University, 88100 Catanzaro, Italy; 3 Electrophysiology Unit, Cardiovascular Department, ARNAS Civico—Di Cristina—Benfratelli Hospital, 90127 Palermo, Italy; lucioteresi@gmail.com; 4 Campus Clinic, University of Barcelona, 08024 Barcelona, Spain; 5 Department of Cardiovascular, Respiratory, Nephrological, Anesthesiological and Geriatric Sciences “Sapienza”, University of Rome, 00161 Rome, Italy; marcoval.mariani@gmail.com (M.V.M.); npierucci@gmail.com (N.P.); 6 Texas Cardiac Arrhythmia Institute, St David’s Medical Center, Austin, TX 78705, USA; vmirco.lafazia@gmail.com; 7 Brain Health Imaging Centre, Centre for Addiction and Mental Health (CAMH), Toronto, ON M6J 1H4, Canada; chiaracamastra@gmail.com; 8 Polimedic, 90127 Palermo, Italy; faziogiova@gmail.com

**Keywords:** atrial fibrillation, vitamin D, 25-hydroxyvitamin D, meta-analysis, systematic review

## Abstract

**Background**: Vitamin D has been implicated in several biological pathways involved in atrial remodeling, yet its association with incident atrial fibrillation (AF) remains uncertain across epidemiological studies. **Objectives**: The objective was to evaluate the association between circulating vitamin D levels and the risk of incident AF through a systematic review and meta-analysis of prospective studies using a harmonized continuous analytical framework. **Methods**: A systematic review and meta-analysis of prospective cohort studies was performed. Hazard ratios (HRs) and 95% confidence intervals (CIs) were pooled using the generic inverse variance method. To improve comparability across studies, effect estimates were harmonized to a common continuous contrast corresponding to a 1-standard-deviation (1-SD) difference in circulating vitamin D levels. Estimates were then oriented such that higher values reflected lower vitamin D status and higher AF risk. Random-effects models were used as the primary analytical approach. Sensitivity analyses included leave-one-out testing and an additional pooled analysis incorporating a threshold-based study after approximate rescaling to the continuous 1-SD framework. **Results**: Five prospective studies were eligible for quantitative assessment. In the primary continuous harmonized analysis, including the four studies with directly reported or more reliably rescaled continuous estimates, lower vitamin D status was associated with a higher risk of incident AF (HR 1.08, 95% CI 1.05–1.11), with no statistical heterogeneity (I^2^ = 0%). In a sensitivity analysis including the HUNT study after approximation of its threshold-based estimate to the continuous 1-SD framework, the pooled association remained statistically significant (HR 1.08, 95% CI 1.05–1.11), with low heterogeneity (I^2^ = 7%). The direction of effect remained stable across sensitivity analyses. **Conclusions**: Lower circulating vitamin D levels were associated with a modestly higher risk of incident AF in prospective studies. The most internally consistent quantitative signal emerged from analyses conducted within a common continuous 1-SD framework. Inclusion of a threshold-based study through approximate rescaling did not materially alter the pooled estimate, although this step relied on additional assumptions. These findings support an observational association but do not establish causality or justify vitamin D supplementation specifically for AF prevention.

## 1. Introduction

Vitamin D deficiency has been associated with an increased prevalence of cardiovascular diseases and a higher risk of mortality due to heart failure, myocardial infarction, atrial fibrillation and sudden cardiac death [1]. Vitamin D deficiency is defined by serum concentrations of 25(OH)D below 21 ng/mL (50 nmol/L) [2], indicative of moderate deficiency, or severe deficiency when levels fall below 10 ng/mL [3,4]. Notably, vitamin D deficiency has been linked to major cardiovascular risk factors, including obesity, insulin resistance, impaired glucose tolerance, diabetes mellitus, arterial hypertension, mixed dyslipidemia, and metabolic syndrome [1]. Atrial fibrillation (AF) is the most common supraventricular arrhythmia and is associated with a significantly increased risk of stroke and heart failure [5,6]. In most cases, AF develops in the context of hypertension, ischemic heart disease, and/or structural ventricular abnormalities [7,8]. More recently, increasing evidence has highlighted the role of genetic predisposition to atrial myopathy and fibrosis as key drivers in the development and maintenance of this arrhythmia [6]. In this context, vitamin D deficiency has been hypothesized to contribute to the pathogenesis of AF through its involvement in inflammation, myocardial remodeling, and modulation of cardiovascular risk factors. Nevertheless, the relationship between vitamin D deficiency and AF remains incompletely understood, with available evidence yielding heterogeneous and sometimes conflicting results. Accordingly, we conducted a systematic review and meta-analysis to evaluate the association between vitamin D deficiency and AF incidence.

## 2. Materials and Methods

This meta-analysis was performed according to the Preferred Reporting Items for Systematic Reviews and Meta-Analyses (PRISMA) guidelines [9] (Table S1). Institutional Review Board approval is not required for this type of study.

This systematic review and meta-analysis was registered in PROSPERO (CRD420261422618).

### 2.1. Search Strategy

Two independent reviewers (L.T. and R.R.) performed a comprehensive and systematic literature search in PubMed, ClinicalTrials.gov, Embase, and the Cochrane Library. The search strategy combined terms related to vitamin D deficiency (“vitamin D deficiency” OR “25-hydroxyvitamin D” OR “25(OH)D”) with terms related to atrial fibrillation (“atrial fibrillation” OR “AF”), in various combinations. Full-text manuscripts published between 1 February 2003 and 31 May 2026 were screened for eligibility.

### 2.2. Study Eligibility

Full-text manuscripts published in peer-reviewed journals investigating the association between vitamin D deficiency and incidence of AF were considered eligible for inclusion. Studies assessing circulating levels of 25-hydroxyvitamin D in relation to the AF incidence (defined as newly diagnosed AF without a previous history of AF, in patients with vitamin D deficiency). Studies reporting the incidence of AF were included. Non–English-language articles, editorials, letters, expert opinions, case reports, or case series, duplicate publications, and meta-analyses were excluded. No restrictions on sample size were applied. Two authors (L.T. and R.R.) independently screened all retrieved studies for eligibility, and any discrepancies were resolved through discussion with a third reviewer (A.C.). Only studies fulfilling all predefined inclusion criteria were included in the final analysis (Table 1).

### 2.3. Data Extraction

The following variables were extracted from each included study: (i) first author, (ii) year of publication, (iii) study design, (iv) sample size, and (v) main demographic and clinical characteristics of the study population. In addition, data on vitamin D status were collected, including circulating levels of 25-hydroxyvitamin D [25(OH)D], as well as the definition and thresholds used to identify vitamin D deficiency. With specific regard to outcomes, information on the AF incidence was recorded. Where available, effect estimates (e.g., odds ratios, hazard ratios, or relative risks) describing the association between vitamin D deficiency and atrial fibrillation were also extracted. The methodological quality of each study was independently assessed using the Newcastle–Ottawa Scale (NOS), with studies categorized as poor, fair, or good quality according to predefined criteria evaluating selection, comparability, and outcome domains [10] (Table S2).

## 3. Statistical Analysis

All statistical analyses were performed using Review Manager (RevMan, version 5.4; Cochrane Collaboration). Hazard ratios (HRs) and 95% confidence intervals (CIs) were used as the common effect measure for incident atrial fibrillation (AF). For meta-analysis, study-specific HRs were transformed into the natural logarithm scale (logHR), and the corresponding standard errors (SEs) were derived from the reported 95% CIs using standard methods. Because the included studies reported the association between circulating 25-hydroxyvitamin D [25(OH)D] and incident AF using different exposure metrics, effect estimates were harmonized to a common continuous contrast corresponding to a 1-standard-deviation (1-SD) increase in circulating vitamin D levels. When a study directly reported an HR per 1-SD increase in 25(OH)D, that estimate was entered without additional scaling. When the original association was reported per fixed-unit increment (for example, per 10 nmol/L increase), the corresponding logHR and SE were rescaled to the 1-SD metric by multiplying both values by the ratio between the study-specific SD and the original unit increment, assuming an approximately log-linear association between vitamin D concentration and AF risk. 

For studies that did not directly provide a continuous estimate but reported broader exposure contrasts, the primary quantitative synthesis preferentially retained studies that could be harmonized more directly within the continuous 1-SD framework. Accordingly, the main pooled analysis included the four studies with directly reported or more reliably rescaled continuous estimates. One additional study (HUNT) reported a threshold-based contrast (<50 vs. ≥50 nmol/L) rather than a directly continuous AF estimate; therefore, it was not included in the primary continuous pooled analysis and was instead incorporated in a sensitivity analysis after approximate rescaling of the published threshold effect to the same 1-SD framework under the log-linearity assumption.

After harmonization to the 1-SD increase metric, all study-specific estimates were re-oriented to ensure a common biological interpretation across studies, such that higher values reflected the association between lower vitamin D status and higher incident AF risk. Operationally, this was achieved by reversing the sign of the study-specific logHR while leaving the SE unchanged [11].

The primary pooled analysis was performed using the generic inverse variance method. Random-effects models were prespecified as the main analytical approach because relevant clinical and methodological heterogeneity across studies was anticipated, including differences in study populations, vitamin D assays, exposure definitions, follow-up duration, AF ascertainment, and covariate adjustment. Fixed-effect estimates were also examined as complementary analyses. Statistical heterogeneity was assessed using Cochran’s Q test and quantified using the I^2^ statistic.

Sensitivity analyses were conducted using a leave-one-out approach, in which the meta-analysis was repeated after sequential exclusion of each study to assess the influence of individual cohorts on the pooled effect estimate and on between-study heterogeneity. An additional sensitivity analysis included the threshold-based HUNT study after approximate continuous rescaling to assess whether its inclusion materially altered the pooled association. For each study, the SE was derived from the reported 95% CI as follow

$$SE=\frac{\ln\left(upperCI\right)-\ln\left(lowerCI\right)}{2*1.96}$$

For studies requiring scale conversion, the rescaled logHR for the 1-SD metric was calculated as:

$$\log(\mathrm{HR}1_{\mathrm{SD}})\;=\;\log({\mathrm{HR}}_{\Delta })\;\times \;\frac{SD}{\Delta }$$

and the corresponding SE was rescaled as:

$$\mathrm{SE}1_{\mathrm{SD}}\;=\;{\mathrm{SE}}_{\Delta }\;\times \;\frac{SD}{\Delta }$$

where Δ represents the original exposure contrast reported by the study and SD, and SD is the study-specific standard deviation of circulating vitamin D, or the best available approximation when direct SD reporting was not available.

Publication bias was not formally tested because of the small number of included studies. Funnel plot inspection, when performed, was considered descriptive only and was not used to support firm conclusions regarding small-study effects.

## 4. Results

### 4.1. Literature Search

**Figure 1:**
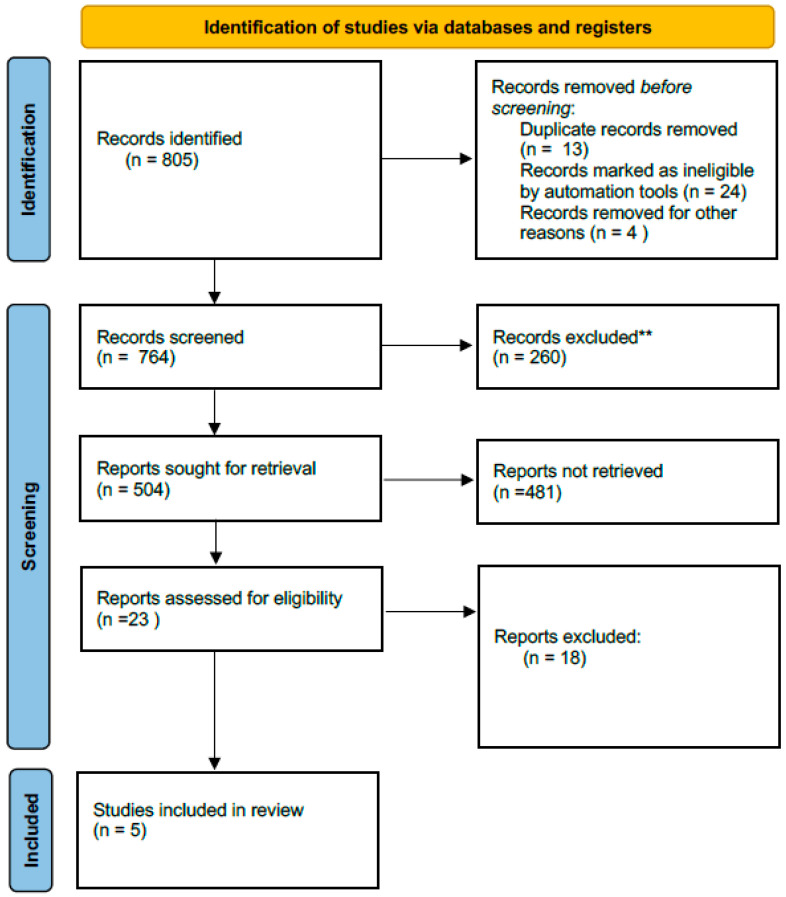
PRISMA 2020 flow diagram illustrating the study selection process for the systematic review. The diagram summarizes the identification, screening, eligibility assessment, and inclusion of studies retrieved from electronic databases and registers. ** Records excluded refers to studies excluded during the title and abstract screening stage. Reasons for exclusion included lack of relevance to the research question, inappropriate study population, intervention/exposure or outcome, unsuitable study design, review articles/editorials/letters, and conference abstracts with insufficient data.

The study selection process is presented in Figure 1. A total of 805 records were identified through database and register searches. Before screening, 13 duplicate records, 24 records marked as ineligible by automation tools, and 4 records removed for other reasons were excluded, leaving 764 records for title and abstract screening.

After screening, 260 records were excluded. The remaining 504 reports were sought for retrieval; 481 reports could not be retrieved, and 23 full-text reports were assessed for eligibility. After full-text evaluation, 18 reports were excluded for prespecified reasons (i.e., study design not meeting inclusion criteria, lack of a prospective evaluation of incident atrial fibrillation, absence of directly usable effect estimates for the association between circulating vitamin D and AF incidence and overlapping or non-eligible populations), and 5 prospective studies were ultimately included in the review and quantitative synthesis [12,13,14,15,16].

The full database-specific Boolean search strings are reported in the Supplementary Material (Table S3).

### 4.2. Study Characteristics

A total of 370,116 participants were included across the five prospective cohort studies considered for quantitative analysis. The study populations were predominantly middle-aged to elderly adults derived from large community-based cohorts, with follow-up durations ranging from approximately 10 to over 20 years.

All included studies assessed circulating 25-hydroxyvitamin D [25(OH)D], although exposure definitions varied. Some studies reported continuous associations per 1-SD or per fixed-unit increment, whereas others reported threshold-based or quantile-based contrasts. AF ascertainment was based on electrocardiographic evidence and/or validated medical records, including hospital discharge diagnoses, registry data, and physician adjudication. Detailed study-level characteristics are presented in Table 1.

The smallest study included 2930 participants [14], while the largest enrolled 348,094 individuals [13]. Follow-up duration ranged from approximately 10 to over 20 years, allowing adequate assessment of incident AF across studies.

### 4.3. Main Analysis

In the primary quantitative synthesis, we included the four prospective studies that reported directly continuous estimates or allowed more reliable rescaling to a common continuous 1-SD framework. After harmonization and orientation to reflect lower vitamin D status and higher AF risk, pooled analysis showed that lower vitamin D levels were associated with a higher risk of incident AF (HR 1.08, 95% CI 1.05–1.11).

**Figure 2:**
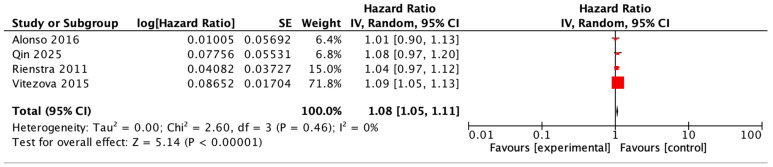
Forest plot of the random-effects meta-analysis evaluating the association between baseline vitamin D deficiency and incident atrial fibrillation. Hazard ratios (HRs) with 95% confidence intervals (CIs) are shown for the individual studies (Vitezova et al. [12], Qin et al. [13], Rienstra et al. [14], and Alonso et al. [15]) and for the pooled estimate. **Abbreviations:** HR, hazard ratio; CI, confidence interval; IV, inverse variance; SE, standard error.

No statistical heterogeneity was observed in this primary pooled analysis (I^2^ = 0%; Cochran’s Q *p* = 0.46) (Figure 2). The harmonized study-specific estimates were closely aligned, with hazard ratios ranging from 1.01 to 1.09. Qin et al. [13] contributed the largest statistical weight, while the remaining studies contributed smaller but directionally concordant effects [12,14,15].

### 4.4. Sensitivity Analysis Including the HUNT Study

Because the HUNT study [16] reported a threshold-based contrast (<50 vs. ≥50 nmol/L) rather than a directly continuous estimate for incident AF, it was incorporated in an additional sensitivity analysis after approximate rescaling of the published threshold effect to the common continuous 1-SD framework.

**Figure 3:**
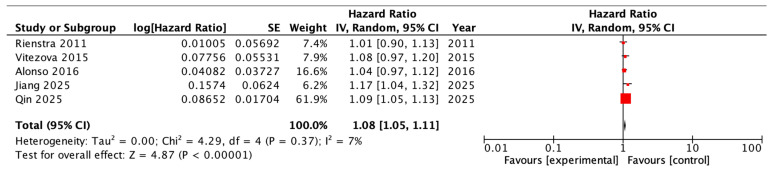
Forest plot of the random-effects meta-analysis evaluating the association between lower baseline vitamin D levels and the risk of incident atrial fibrillation. Hazard ratios (HRs) with 95% confidence intervals (CIs) are shown for the individual studies (Rienstra et al. [14], Vitezova et al. [12], Alonso et al. [15], Jiang et al. [16], and Qin et al. [13]) and for the pooled estimate.

In this expanded five-study analysis, the association between lower vitamin D status and incident AF remained statistically significant (HR 1.08, 95% CI 1.05–1.11), with low heterogeneity (I^2^ = 7%) (Figure 3). Thus, inclusion of the threshold-based study did not materially alter the pooled effect estimate, although it introduced a small increase in between-study heterogeneity (Figure 3).

### 4.5. Sensitivity Analyses

Leave-one-out sensitivity analyses were performed to evaluate the influence of each individual study on the pooled estimate. In the primary four-study continuous analysis, sequential exclusion of individual studies did not materially change the direction or statistical significance of the pooled association. The overall findings therefore appeared stable within the continuous harmonized framework (Table 2).

### 4.6. Publication Bias and Grading of Evidence

Given the small number of included studies, publication bias was not formally assessed. Any funnel plot inspection was considered descriptive only and was not interpreted as evidence for or against small-study effects.

According to the GRADE Working Group framework [17], the overall certainty of evidence for the association between lower vitamin D levels and incident AF was rated as moderate (Table S4). This rating reflects the observational nature of the included studies, which inherently limits causal inference, despite the high methodological quality of the cohorts and the consistency of findings across analyses.

## 5. Discussion

In this systematic review and meta-analysis of prospective cohort studies, lower circulating vitamin D levels were associated with a modestly higher risk of incident atrial fibrillation. The most internally consistent quantitative signal emerged from the primary analysis restricted to studies that could be harmonized more directly within a continuous 1-SD framework. Under this approach, the pooled estimate was statistically significant, and no statistical heterogeneity was detected.

A key strength of the present work is that it distinguishes between studies that could be incorporated more directly into a common continuous analytical framework and those that require broader approximation. Specifically, the main pooled result was derived from four studies with directly reported or more reliably rescaled continuous estimates. This strategy reduced scale-related incompatibility across exposure definitions and yielded a coherent summary estimate with no detectable statistical heterogeneity. Importantly, when the HUNT study was additionally incorporated through approximate rescaling of its threshold-based estimate to the same 1-SD framework, the pooled effect remained essentially unchanged, and heterogeneity increased only slightly. This pattern suggests that the overall association is not dependent on the exclusion of a single eligible study, while also confirming that threshold-based data are methodologically less straightforward to incorporate into a continuous model.

Qin et al. [13] contributed the largest statistical weight in the primary pooled analysis, reflecting the very large size of the UK Biobank cohort. Accordingly, the pooled estimate should not be interpreted as a simple arithmetic summary of equally informative studies. Nevertheless, the remaining studies within the primary continuous harmonized framework were directionally concordant, suggesting that the observed association was not contradicted by the smaller cohorts.

From a biological perspective, the observed association is plausible. Vitamin D has been implicated in pathways relevant to atrial structural and electrical remodeling, including inflammation, renin–angiotensin–aldosterone system modulation, myocardial fibrosis, and broader cardiometabolic regulation [18,19,20,21,22]. These mechanisms provide a conceivable basis for a link between lower vitamin D status and AF development [23]. However, biological plausibility should not be confused with proof of causation.

Residual confounding remains a major limitation of the observational evidence. Low vitamin D status is strongly associated with obesity, lower physical activity, diabetes mellitus, chronic kidney disease, poorer nutritional status, and reduced sunlight exposure [24,25,26]. Even when individual studies adjusted for many of these variables, adjustment strategies differed substantially across cohorts, and unmeasured or residual confounding cannot be excluded.

This distinction is clinically important. The present findings support an observational association but do not justify the inference that vitamin D supplementation prevents AF. Randomized interventional evidence has not consistently shown a preventive effect of vitamin D supplementation on incident atrial fibrillation. Accordingly, the current results should not be interpreted as supporting routine vitamin D supplementation specifically for AF prevention [27]. Further studies are warranted to determine whether targeted correction of vitamin D deficiency could have a role in AF prevention in selected high-risk groups.

Our findings should be interpreted in the context of prior meta-analyses investigating the association between vitamin D and incident AF [15,28]. Alonso et al. [15] was eligible as an original prospective cohort study and was therefore included in the present quantitative synthesis, whereas Ding et al. [28] was excluded because it was a secondary meta-analysis rather than a primary study. Compared with previous reviews, the present work offers a more focused synthesis of prospective cohort data, but it also remains limited by the small number of eligible studies and the strong statistical contribution of the largest cohort.

Although no statistical heterogeneity was detected in the primary continuous pooled analysis, this should not be interpreted as evidence of full clinical comparability across studies. Important differences remained in vitamin D assay methods, exposure definitions, follow-up duration, AF ascertainment, participant characteristics, and adjustment strategies. Thus, statistical homogeneity within the harmonized framework does not eliminate underlying clinical and methodological heterogeneity.

## 6. Limitations and Strengths

The present study should also be interpreted in light of its methodological limitations. First, only a small number of prospective studies were available for quantitative synthesis. Second, despite harmonization, the included studies differed in population characteristics, exposure definitions, laboratory assays, follow-up duration, and covariate adjustment. Third, one study required approximate rescaling from a threshold-based contrast to the continuous 1-SD framework, which necessarily relied on additional assumptions, particularly approximate log-linearity. Fourth, formal assessment of publication bias was not reliable because of the limited number of included studies.

Despite these limitations, the study has important strengths. It focused specifically on prospective cohort studies, thereby improving temporal coherence between vitamin D exposure and incident AF. It also adopted a transparent harmonization strategy and clearly separated the primary continuous pooled analysis from the additional sensitivity analysis incorporating threshold-based data. This approach improves interpretability and better reflects the true evidentiary structure of the available literature.

## 7. Conclusions

Lower circulating vitamin D levels were associated with a modestly higher risk of incident atrial fibrillation in prospective studies. The most internally consistent quantitative evidence emerged from analyses conducted within a common continuous 1-SD framework. Inclusion of a threshold-based study through approximate rescaling did not materially alter the pooled estimate, although this step relied on additional assumptions. Overall, these findings support an observational association but do not establish causality or support vitamin D supplementation specifically for AF prevention.

## Figures and Tables

**Table 1:** Patients’ baseline characteristics.

First Author	Year	Study Design	Sample Size	Age (Years)	Male (%)	Hypertension (%)	Diabetes (%)	Dyslipidemia (%)	Vit D Deficiency (%)	Vitamin D Definition (Cut-Off)	AF Ascertainment Method	FOLLOW-UP	AF Events (n, %)
**Rienstra**	2011	Prospective cohort (Framingham)	2930	65 ± 9	55	49%	8%	NR	NR	Continuous (per SD of 25(OH)D)	ECG + medical records + adjudication	Mean 9.9 years	425 (14.5)
**Vitezova**	2015	Prospective cohort (Rotterdam Study)	3395	69 ± 9	43	60%	12%	NR	57% (<50 nmol/L)	<50 (deficient), 50–75 (insufficient), ≥75 nmol/L	ECG + medical records + GP records	Median 12 years	263 (7.7)
**Alonso**	2016	Prospective cohort (ARIC)	12,303	56 ± 6	45	37%	NR	NR	32% (<20 ng/mL)	<20, 20–<30, ≥30 ng/mL	ECG + hospital discharge codes + death certificates	Median 21.4 years	1866 (15.2)
**Jiang**	2025	Prospective cohort (HUNT)	3394	62 ±11	47	44.8%	4.7%	27.1	44% (<50 nmol/L)	<50 vs. ≥50 nmol/L (mean of 2 measurements over 10 years)	Hospital registry + physician validation (ECG-based)	Median 12.1 years	304 (9)
**Qin**	2025	Prospective cohort (UK Biobank)	348,094	56 ±8	46	NR	NR	NR	NR (quartiles)	Quartiles (Q1–Q4, highest vs. lowest)	Hospital records + ICD codes	Median 12 years	21,230 (6.1)

Categorial variables are given as absolute numbers and percentages, n (%). Continuous variables are given as mean ± standard deviation. Legend. AF: atrial fibrillation, 25(OH)D: 25-hydroxyvitamin D, NR: not reported.

**Table 2:** Leave-one-out analysis for AF incidence.

Excluded Study	Effect Estimate	*p*-Value	I^2^ (%)
Rienstra et al. [14]	5.71	<0.001	0
Vitezova et al. [12]	3.35	<0.001	30
Alonso et al. [15]	5.10	<0.001	3
Qin et al. [13]	2.26	0.02	16

## Data Availability

All data generated or analyzed during this study are included in this published article and its Supplementary Information files.

## Supplementary Materials

The following supporting information can be downloaded at https://www.mdpi.com/article/10.3390/biomedicines14071580/s1. Table S1: PRISMA 2020 Checklist; Table S2: The Newcastle-Ottawa Scale (NOS) for assessing the quality of studies in meta-analyses; Table S3: Search Strategy; Table S4: Grading of evidence for the association of AF incidence in patients with vitamin D deficiency.

## Abbreviations

The following abbreviations are used in this manuscript:

AF	Atrial Fibrillation
CI	Confidence Interval
GRADE	Grading of Recommendations Assessment, Development and Evaluation
HR	Hazard Ratio
I^2^	Higgins’ Inconsistency Statistic
NOS	Newcastle–Ottawa Scale
PRISMA	Preferred Reporting Items for Systematic Reviews and Meta-Analyses
Q	Cochran’s Q Statistic
RevMan	Review Manager
SE	Standard Error
SD	Standard Deviation
25(OH)D	25-Hydroxyvitamin D
