# Supplementary material for: Association Between Vitamin D Deficiency and the Incidence of Atrial Fibrillation: A Systematic Review and Meta-Analysis
Source: Biomedicines. 2026 Jul 15;14(7):1580. doi: 10.3390/biomedicines14071580 (PMC13407350; doi:10.3390/biomedicines14071580)
Supplement: Supplementary file 1 [file biomedicines-14-01580-s001.zip › Table S3.pdf]

Table S3. SEARCH strategy

(("vitamin D deficiency"[Title/Abstract]
OR "25-hydroxyvitamin D"[Title/Abstract]
OR "25(OH)D"[Title/Abstract]
AND
("atrial fibrillation"[Title/Abstract]
OR AF[Title/Abstract]))
