# Supplementary material for: Association Between Vitamin D Deficiency and the Incidence of Atrial Fibrillation: A Systematic Review and Meta-Analysis
Source: Biomedicines. 2026 Jul 15;14(7):1580. doi: 10.3390/biomedicines14071580 (PMC13407350; doi:10.3390/biomedicines14071580)
Supplement: Supplementary file 1 [file biomedicines-14-01580-s001.zip › Table S4.pdf]

Table S4. Grading of evidence for the association of AF incidence in patients with vitamin D deficiency.

Certainty assessment							Certainty
**Number of studies**	**Study design**	**Risk of bias**	**Inconsistency**	**Indirectness**	**Imprecision**	**Other considerations**	
**AF incidence**							
5	Observational cohort studies	Some concern	No	Some concern	Some concern	No pubblication bias	⊕⊕⊕◯ Moderate

Legend. AF: atrial fibrillation
