# Supplementary material for: Association Between Vitamin D Deficiency and the Incidence of Atrial Fibrillation: A Systematic Review and Meta-Analysis
Source: Biomedicines. 2026 Jul 15;14(7):1580. doi: 10.3390/biomedicines14071580 (PMC13407350; doi:10.3390/biomedicines14071580)
Supplement: Supplementary file 1 [file Table S2.pdf › Table S2.pdf]

Table S2. The Newcastle-Ottawa Scale (NOS) for assessing the quality of studies in meta-analyses

**Studies and year**	**Selection**				**Comparability**	**Outcomes**			**Total**
	Representativeness of the exposed cohort	Selection of the non exposed cohort	Ascertainment of exposure	Demonstration that outcome of interest was not present at start of study	Study controls for cardiac reverse remodeling	Assessment of outcome	Was follow-up long enough for outcomes to occur	Adequacy of follow up of cohorts	Maximum 9
Rienstra et al 2011	1	1	1	1	2	1	1	1	9
Vitezova et al 2015	1	1	1	1	2	1	1	1	9
Alonso et al 2016	1	1	1	1	2	1	1	1	9
Jiang et al 2025	1	1	1	1	1	1	1	1	8
Qin et al 2025	1	1	1	1	1	1	1	1	8
